# Primary eye care through vision centres—key to universal eye health

**Published:** 2022-03-01

**Authors:** Thulasiraj Ravilla

**Affiliations:** 1 Executive Director: LAICO-Aravind Eye Care System, Madurai, India.

**Primary eye care through technology-enabled vision centres is a key strategy to achieve universal eye health.**

One of the many reasons that primary eye care is gaining importance is that it is recognised as a necessary component of primary health care. In 2015, member nations of the World Health Organization (WHO) set universal health coverage as one of the targets of the Sustainable Development Goals (SDG).^1^ Primary health care was advocated as a key strategy for achieving universal health coverage. This holds good for eye health too. In India, primary eye care through vision centres is emerging as a crucial measure for achieving universal eye health.^2^ Primary eye care is by design a locally delivered service, which can address issues of accessibility and associated costs that have surfaced as serious barriers during the COVID-19 pandemic.

## The need for accessible eye care

The costs associated with transport, lost wages, and the need for an attendant to accompany a patient reduce the affordability of eye care, adversely impacting health-seeking behaviour. Eye camps have been a means of providing accessible and affordable primary eye care in areas where there is little or no eye care. While the camp approach continues to be critical to delivering eye care to rural areas, its reach is quite limited and lacks sustainability, given the inherent limitation of eye camps being held for just a day or so in any given location.^3^ Also, the current COVID-19 pandemic and events like elections cause disruption, reinforcing the need for an alternative provision for accessible eye care.

**Figure 1:**
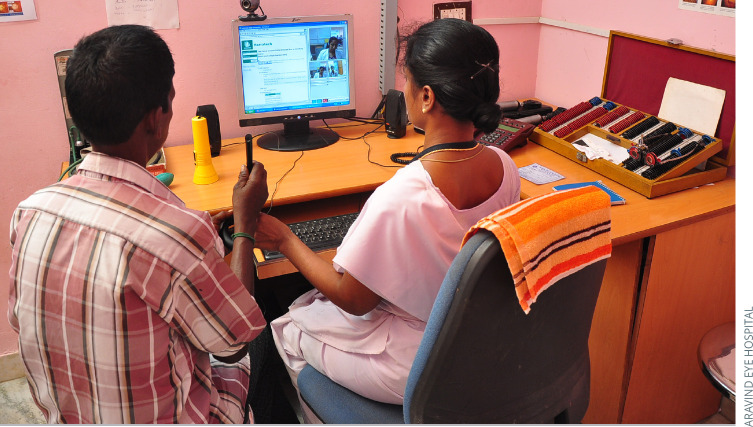
Aravind vision centre at Alanganallur, Madurai. **INDIA**

In a population of around 50,000, a significant proportion would be 40 years or older, many of whom would have an eye care need. It is likely that 25 to 30% of this population would need some eye care—i.e., almost everyone above 45 years of age, constituting over 25% of the population,^4^ will need eye care, and some under 45 too. Thus, a vision centre is viable for a population base of 50,000 (Figure 1). Depending on the situation, a vision centre can be a standalone facility or an integral part of an existing health care facility. A population of 50,000 in the context of South Asian countries would likely live within a radius of 10 km and thus find the facility accessible. Locating the vision centre in a rural town with public transport connectivity will further increase accessibility. This alone can positively impact health-seeking behaviour.

## Scope of primary eye care

In addition to accessibility, another component of universal health coverage is the comprehensiveness of care. Trained ophthalmic technicians can provide comprehensive eye care at vision centres. It is also possible to provide comprehensive care with the use of technology and through referral linkages.

To do this well, we have to define the scope of services, as suggested in Table 1.

**Table 1:** Scope of services

	**Case finding**	**Intervention**			**Compliance/follow up**
		**Curative**	**Prevention**	**Referral**	
**Cataract**	**✓**			**✓**	**✓**
**Refractive error**	✓	✓			✓
**Childhood blindness**	✓		✓	✓	✓
**Diabetic retinopathy**	✓*			✓	✓
**Glaucoma**	✓*			✓	✓
**Corneal aberrations, ulcers, etc.**	✓	✓	✓	✓	✓
**Trachoma**	✓			✓	✓
**Low vision**	✓			✓	✓

* Using low-cost imaging and technologies like artificial intelligence (AI)

The essential equipment in a vision centre should ideally include visual acuity charts, a trial set and retinoscope for refraction, a slit lamp for a detailed eye examination, an ophthalmoscope for fundus examination, a tonometer for intraocular pressure measurement, a glucometer, a weighing machine and height chart, as well as thermometer and blood pressure apparatus to measure vital signs. Vision centre staff are trained to carry out comprehensive eye examinations using the equipment mentioned above.^5^ In addition, a computer with internet connectivity can facilitate the practice of telemedicine, computerising of medical records, and performance of administrative tasks, thereby enhancing the quality and efficiency of vision centres. A low-cost fundus camera with a clear protocol for its use, supported by telemedicine, can further enhance the comprehensiveness of diagnosis.

In addition to centre-based activities, vision centres can support community-based activities such as school screening. Vision centres, by virtue of their easy accessibility, have the potential to enhance adherence to treatment in patients with chronic conditions, such as glaucoma or diabetic retinopathy, as well as follow up on patients who have been advised to have surgery or referred to a hospital.

While rural vision centres are uniquely positioned to contribute to universal eye health, the reality is that many ophthalmologists and administrators are reluctant to work in small rural settings. Hence services would necessarily have to be delivered by an ophthalmic technician, supported by a similar cadre of staff taking care of administrative tasks. In this context, the service design and implementation will need to incorporate the following to achieve universal eye health:

- In the absence of an ophthalmologist, neither comprehensive eye examination nor the quality of diagnosis or care should be compromised. Telemedicine and electronic medical records can be very useful in providing quality eye care. In the context of the COVID pandemic, safer methods need to be adopted for completing comprehensive eye examination.^6^
- The care cycle should be completed—i.e., patients visiting the vision centre should receive a diagnosis if possible and treatment advice. They should be able to obtain spectacles and medicines as prescribed, or get help to visit an eye hospital for surgery or advanced management when indicated.
- All of this has to be affordable for the community. Where possible, each vision centre should also be able to sustain its own operating costs financially. This can happen through charging affordable consulting fees and making margins from the sale of medicines and spectacles, in the process saving patients money and effort.

## Monitoring

Integral to effective implementation is a robust monitoring system. Given the potential of vision centres to support the delivery of universal eye health, the success of a vision centre should be measured by comparing the number of people receiving eye care with the estimated number of people in the population or community who need eye care services (the denominator). Table 2 gives a rough estimate of eye care needs for 50,000 people, a typical population base for a vision centre. This estimate can be refined for each centre based on actual details. The annual performance and coverage should be benchmarked against this ‘denominator’.

**Table 2:** Eye care needs for a population of 50,000*

**Eye care needs for 50,000 population**	**Estimate**
Those in need of any form of eye care (25% of the population)	12,500
**Annual need**	
Cataract surgeries (based on cataract surgical rate, CSR, of 10,000)	500
Spectacles (20%; and a change in 5 years)	2,000
**Ongoing care**	
Patients with glaucoma (1%)	500
Patients with diabetes (3%)	1,500
Incurably blind and those with low vision (each at 0.08%)	40+40

* This number serves as an illustration. The population base or the assumed rates can be changed for various conditions based on local evidence.

The monitoring system should also monitor adherence to treatment or referral. Ultimately, it is the adherence to prescribed treatment or referral that will help manage eye health conditions. Ongoing monitoring and continuous review will result in improving eye care.

## Conclusion

Well-designed primary eye care approaches, such as technology-enabled vision centres, have the potential to contribute to the achievement of universal eye health. Since such an approach to delivering primary eye care mitigates the problem of accessibility, it may be less affected by factors such as the continuing COVID-19 pandemic. It was seen that once the Covid-19 travel restrictions were lifted in India in September 2020, most vision centres in our network, being at an easily reachable distance, reported that their patient volumes were back to normal much sooner than in secondary and tertiary eye hospitals.

## References

[1] World Health Organization. SDGs and progress towards universal health coverage. Regional Office for South-East Asia: World Health Organization; 2017. Available from: https://apps.who.int/iris/handle/10665/258544 (accessed 16 January 2021).

[2] World Health Organization. Universal eye health: a global action plan 2014–2019. Geneva: World Health Organization; 2013. Available from: www.iapb.org/learn/resources/who-global-action-plan-2014-2019 (accessed 23 January 2021).

[3] Fletcher AE Donoghue M Devavaram J Thulasiraj RD Scott S Abdalla M, et al. Low uptake of eye services in rural India: a challenge for programs of blindness prevention. Arch Ophthalmol. 1999;117(10):1393-9. 10532449 10.1001/archopht.117.10.1393

[4] Office of the Registrar General & Census Commissioner. Census of INDIA 2001—population projections for India and states 2001–2026: report of the technical group on population projections constituted by the National Commission on Population May 2006. New Delhi. Available from: https://bit.ly/3xjiKOz (accessed 12 July 2021).

[5] Khanna RC Sabherwal S Sil A Gowth M Dole K Kuyyadiyil S, et al. Primary eye care in India—the vision center model. Indian J Ophthalmol. 2020;Feb;68(2):333-9. Available from: doi: 10.4103/ijo.IJO_118_19 (accessed 12 July 2021). 10.4103/ijo.IJO_118_19 PMC7003605 31957722

[6] Vashist P Senjam SS Gupta V Manna S Agrawal S Gupta N, et al. Community eye-health and vision centre guidelines during COVID-19 pandemic in India. Indian J Ophthalmol. 2020;68:1306-11. 32587155 10.4103/ijo.IJO_1527_20 PMC7574078

